# An Assessment of the Intestinal Lumen as a Site for Intervention in Reducing Body Burdens of Organochlorine Compounds

**DOI:** 10.1155/2013/205621

**Published:** 2013-02-07

**Authors:** Ronald J. Jandacek, Stephen J. Genuis

**Affiliations:** ^1^Department of Pathology and Laboratory Medicine, University of Cincinnati, 2120 East Galbraith Road, Cincinnati, OH 45237, USA; ^2^Faculty of Medicine, University of Alberta, 2935-66 Street, Edmonton, AB, Canada T6K 4C1

## Abstract

Many individuals maintain a persistent body burden of organochlorine compounds (OCs) as well as other lipophilic compounds, largely as a result of airborne and dietary exposures. Ingested OCs are typically absorbed from the small intestine along with dietary lipids. Once in the body, stored OCs can mobilize from adipose tissue storage sites and, along with circulating OCs, are delivered into the small intestine via hepatic processing and biliary transport. Retained OCs are also transported into both the large and small intestinal lumen via non-biliary mechanisms involving both secretion and desquamation from enterocytes. OCs and some other toxicants can be reabsorbed from the intestine, however, they take part in enterohepatic circulation(EHC). While dietary fat facilitates the absorption of OCs from the small intestine, it has little effect on OCs within the large intestine. Non-absorbable dietary fats and fat absorption inhibitors, however, can reduce the re-absorption of OCs and other lipophiles involved in EHC and may enhance the secretion of these compounds into the large intestine—thereby hastening their elimination. Clinical studies are currently underway to determine the efficacy of using non-absorbable fats and inhibitors of fat absorption in facilitating the elimination of persistent body burdens of OCs and other lipophilic human contaminants.

## 1. Introduction

The presence of organochlorine (OC) compounds in the environment and biosphere is a recent development in evolutionary terms. The intentional and inadvertent synthesis and widespread dispersion of OCs began in the twentieth century. Compounds including hexachlorobenzene, DDT, and PCBs were produced to meet industrial needs. Other OCs, including dioxins, have been produced as industrial byproducts. In addition to OCs, other lipophilic compounds including brominated hydrocarbons (to be used principally as flame retardants) have also been produced.

The magnitude of the industrial output and the chemical stability of OC compounds have resulted in their persistence in the environment. Even with planned reductions in the production and use of these compounds, they will remain in the environment for many decades. 

The ubiquitous presence of OCs has resulted in their entry into the food chain, with accumulation in higher organisms. Their lipophilicity directs them to be stored in adipose depots of animals, including humans. Many OCs and their metabolites exit the body very slowly, resulting in long-storage half-lives. 

There is a large body of evidence linking elevated levels of OCs to the risk of disease such as diabetes and hypertension [1, 2]. Some OCs are considered to be carcinogens in animals and humans. Although effort is underway to reduce the exposure of people to OCs, this work toward decreasing environmental levels will presumably not produce a significant reduction in the levels in the biosphere in the immediate future and does not address the bioaccumulated burden already present in many individuals. 

Given the persistent and ubiquitous nature of OCs, and given the potential link to the risk of disease, it is desirable to consider strategies to reduce their level in the body. This paper considers the intestinal lumen as a site for intervention to reduce human exposure and the resulting detrimental effects on health. 

It is generally thought that most of human exposure to OCs comes through ingestion of foods that contain OCs. Undoubtedly there are many situations in which entry is by inhalation or by the transdermal route, but food-borne OCs dominate the entry route for most people. It is clear that the interruption of absorption of OCs from the intestinal lumen into the systemic circulation can reduce the accumulation of OCs in the body.

It is also clear that many OCs undergo enterohepatic circulation; that is, they move from tissues in which they are stored to the blood, are taken up by the liver, and enter the intestine in bile. They may also enter the intestine directly from cells that line the intestinal lumen. Reabsorption from the intestine into systemic blood completes the enterohepatic circulation loop. As in the case for dietary OCs, inhibition of the reabsorption step can direct OCs to the large intestine where they will be excreted in the feces.

**Figure 1:**
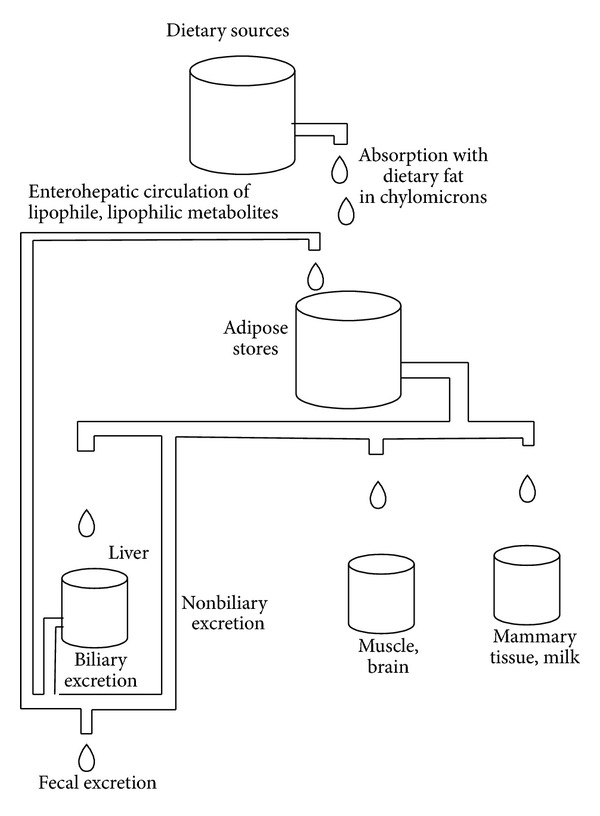
A schematic view of the entry, storage, and excretion of organochlorine compounds.

In the remainder of this paper, we discuss the means by which absorption from the intestinal tract might be reduced. We also discuss how this intervention might reduce the body's stores of OCs and comment on some considerations relating to clinical care. A schematic view of the involvement of the intestine in OC metabolism is presented in Figure 1.

## 2. Oral Absorption

For most people, the entry of OCs into the body is via oral ingestion. Their absorption is therefore from the lumen of the small intestine. Based on measurements of the level of OCs as a function of age, there is evidence that some people take in OCs more rapidly than they excrete them from the body [3, 4]. Reducing the intake of OCs from the diet is therefore a strategy that should be considered in high risk situations in light of the fact that the food chain will continue to contain OCs even if their production rates are curtailed.

Most OCs are very lipophilic, with partition coefficients, expressed as the log_10_ of the ratio of solubility in octanol to that in water of >6.0 [5]. This lipophilicity results in solubility in food lipids, and the presence of dietary triacylglycerol fat acts to facilitate the absorption of OCs. Dietary lipophilic OCs accompany triacylglycerol through absorption via the lymphatic route with little absorption into the portal vein [6]. They are incorporated into chylomicrons, and in this form a portion of the absorbed OC is delivered to peripheral tissues without encountering the liver for first pass metabolism. 

It is possible to reduce of the absorption lipophiles from the intestine. Cholesterol absorption can be reduced by the addition of plant sterols to the diet or by ezetimibe, which blocks the internalization of the cholesterol NPC1L1 complex. This approach is specific for cholesterol and has little effect on other dietary lipophiles.

The addition of a nonabsorbable oil to the diet also hinders the absorption of dietary cholesterol. Olestra, which is comprised of sucrose bonded by ester links to 6–8 long-chain fatty acids, is not absorbed from the intestine. Its ingestion results in an intestinal lipophilic solvent sink that carries a portion of other dietary lipophiles into the large intestine and the feces. Olestra has been reported to significantly reduce the absorption of dietary lipophiles including cholesterol, retinol, vitamin A, vitamin D, vitamin E, vitamin K, *β*-carotene, and lycopene [7]. 

Olestra also interferes with the absorption of OCs. Carbon-14-labeled DDT dissolved either in soybean oil or a 50-50 blend of soybean oil, and olestra was given by gastric gavage to rats that had been surgically fitted with a cannula in the thoracic duct [8]. The percent of ^14^C-DDT dosed in soybean oil that appeared in the lymph by the animals that received soybean oil was 66.6 ± 1.9, while the percentage recovered in the lymph of the animals that received the olestra-soybean oil blend was 21.0 ± 2.4.

Reductions in the absorption of two ^14^C-labeled PCB congeners were also observed when olestra was included in the diet [9]. Six fasted mice were given a dose of either 2,2′,5,5′-tetrachlorobiphenyl or 3,3′,4,4′-tetrachlorobiphenyl in safflower oil. They then received a diet that contained either 19% (weight) butter fat, or the same diet to which 10 g of olestra was added for each 100 g of diet. Complete fecal collections were made for 48 hours after the gavage, and radioactivity was assayed. Based on these excretion data, the absorption of 2,2′,5,5′-tetrachlorobiphenyl was 81.0 ± 1.4% of the dose when the butter-based diet was fed, and 55.2 ± 4.3% when the diet contained olestra. Similarly, the absorption of 3,3′,4,4′-tetrachlorobiphenyl was 43.7 ± 2.0% during the butter-based diet, and 16.5 ± 4.7% with the diet containing olestra.

From the perspective of minimizing the body burden of OCs, an argument can be made that the most effective mean is that of reducing OC intake. Ideally this approach would be that of removing all OCs from the environment and food. Given the difficulty in reducing residual OCs in the environment, a reduction in intake by hindrance of intestinal absorption is an option that may be considered. The decrease in the absorption of the two PCB congeners noted above corresponds to reductions of 33% and 64% of that ingested. Whether that range of reduction is clinically meaningful depends on background intake of OCs, which is generally unknown for most people. However, in geographical area of known contamination, a prophylactic approach that reduces absorption could be beneficial.

The current state of knowledge does not allow us to determine an effective minimum dose of olestra to significantly reduce the absorption of OCs. Ingestion of 14 g/day of olestra significantly reduced the absorption of dietary cholesterol by 16% [10]. Only insignificant reductions in blood tocopherol levels were reported at levels of 10 and 20 g/day of olestra [11]. 

The consideration of the absorption of OCs in the diet is important since the ubiquitous nature of OCs makes it essentially impossible to completely exclude them from our diet even in geographical areas that have not been subject to extensive contamination. Also OCs that enter the intestine from the diet are in the same milieu as OCs and their lipophilic metabolites in enterohepatic circulation which enter the intestine in bile and by nonbiliary transport, as will be discussed below. 

## 3. Enterohepatic Circulation

Enterohepatic circulation is a well-documented process that is generally associated with bile acid metabolism. Bile acids enter the intestinal lumen in bile, help to emulsify and solubilize dietary lipids and their digestion products, and then are actively reabsorbed in the distal part of the small intestine. This reabsorption is an efficient process that returns 90–95% of luminal bile acids back to the liver and ultimately to bile. This recycling, termed enterohepatic circulation, conserves the body's bile acid pool with only a small portion being fecally excreted with each of approximately 12 cycles per day. 

There is evidence that other compounds undergo a similar enterohepatic circulation. This evidence includes the use of olestra in the diet and the measurement of fecal excretion. The first study of olestra in enterohepatic circulation was with cholesterol [12]. Rats were intravenously injected with ^14^C-labelled cholesterol and then fed diets with or without olestra. Excretion of radioactivity in the neutral sterol fraction of feces was significantly higher in the animals fed olestra. Since the cholesterol had been given systemically, the excreted sterol had entered the intestine in bile or possibly directly through enterocytes, and the introduction of olestra slowed its reabsorption.

A similar study was carried out with ^14^C-labelled DDT in gerbils [13]. The animals were dosed with DDT in corn oil, and fecal excretion of radioactivity was followed for 3 months. Olestra was provided in the diet at levels of 2.5, 5.0, and 10.0% by weight. Relative to the control group, increases of 2-3 fold in the rate of DDT excretion were seen in the olestra group. When a regimen of caloric restriction was included, the animals that received 10% olestra increased DDT excretion by 8-fold. Two weeks of this latter treatment resulted in a 50% reduction in total body burden. The data were consistent with the interruption of the enterohepatic circulation of DDE, the principal metabolite of DDT. Moreover, the data indicated that a regimen of olestra and caloric restriction would provide a clinically meaningful reduction in the body burden of an OC.

There is evidence that a significant part of nondietary OCs that enter the lumen of the small intestine is not in bile [14]. The mode of this transport is poorly understood but presumably involves sloughing of enterocytes and direct exudation of the OC from the enterocyte into the lumen. It seems reasonable to assume that OCs from intestinal cells enter the same pool in the lumen as OCs that enter in bile, and that they are absorbed from the intestine after dissolution in micellar forms. This kind of nonbiliary entry into the lumen of the intestine has also been seen for cholesterol although this process may be specifically related to ABCG5/G8 transporters [15]. 

The combination of olestra with weight loss was also studied in mice with body burdens with ^14^C-labelled hexachlorobenzene (HCB) [16]. Caloric restriction combined with olestra resulted in a 30-fold increase in the rate of excretion of HCB. 

Evidence that olestra can enhance the removal of dioxins from humans was presented by Geusau et al [17]. They found that daily ingestion of 15–66 g of olestra increased the excretion rate of 2,3,7,8-tetrachlorodibenzo-*p*-dioxin by 8–10 fold. During the 38 days of this regimen, the patients did not experience gastrointestinal side effects. Based on these results, the use of olestra for the treatment of chloracne associated with dioxins was proposed by Sterling and Hanke [18].

Moser and McLachlan studied the effect of olestra on the excretion of PCBs, polychlorinated dibenzo-p-dioxins, and dibenzofurans in 4 healthy human volunteers who did not have a history of excessive exposure to these compounds [19]. The participants ate 25 g of olestra for each of 3 days, and fecal excretion of the OCs was measured. For essentially all comparisons with the nonolestra period, marked increases in the daily excretion rate were observed. The increases were 1.5–11-fold relative to the nonolestra period. PCB 153, for example, was excreted 6–10 times as rapidly during the olestra period. The excretion of this congener was 65–270 ng/d during the control period and 560–1790 ng/day during the olestra period.

These latter data allow us to estimate whether the inclusion of olestra can have a clinically meaningful effect on the body burden of OCs such as PCBs in a period of time that is practical for patient compliance. We can combine the data from Moser and MacLachlan with the known levels of PCBs in the population. As noted above, the relatively highly chlorinated congener, PCB 153, was studied in their trial and was also measured as a part of the National Health and Nutrition Examination Survey (NHANES) as recently as 2004 [20]. A 70 kg person with 20% body fat would have an adipose depot of 14000 g. We can estimate the concentration of PCBs in the adipose tissue from the lipid-adjusted concentration in serum. For a male in the 50th percentile in the NHANES data, this value is 19.7 ng/g (ppb), and the total amount of PCB 153 in the body would be 14000 g multiplied by 19.7 ng/g, which is equal to 0.28 mg. This rate of excretion corresponds to 8.2% of the body burden being excreted in a year and a half-life of 81 years. 

Moser and McLachlan reported that olestra increased the rate of excretion of PCB 153 by factors ranging from 5.6 to 8.6. If the rate of excretion of PCB 153 is increased 5-fold, the excretion rate would be 43% of the body burden in a year with a corresponding half-life of 1.6 years. The results of these hypothetical calculations are consistent with clinically significant effects of intervention with olestra to reduce body burdens of OCs. Although this kind of calculation can be instructive, it does not answer questions about the variability in an individual's metabolism of OCs that are relevant to the practicality of a regimen that interferes with their enterohepatic circulation. 

## 4. Mechanisms by Which Fecal Excretion Is Enhanced by Nonabsorbable Lipid

Two mechanisms have been proposed for the way that a nonabsorbable lipid enhances fecal excretion of stored OCs: (1) “using the gut wall as a dialysis membrane” [21] and (2) partitioning of OC in the lumen of the intestine into the nonabsorbable lipid [22]. Although both mechanisms may be valid, there are considerations that argue against the importance of the “dialysis” mechanism in the small intestine.

The “dialysis” mechanism is one in which the nonabsorbable lipid makes direct contact with the mucosal enterocytes and extracts OCs through and from the membrane of the intestinal cells. There are three principal reasons that argue against this mechanism as a major factor. First, if the enhancement of secretion through the cell wall results from contact with intestinal fat, then a normal diet would be expected to induce the same process. A typical diet in the US contains 70–100 g of triacylglycerol, a mass that is 3–5-fold times the mass of olestra that has been used in enhancing OC excretion [23, 24]. This fat is hydrolyzed rapidly into fatty acids and monoacylglycerol, but it enters the duodenum mostly as intact triacylglycerol. 

A second reason to reject the idea that OCs are extracted from the enterocyte in the small intestine is the understanding of the barrier of the unstirred water layer that covers the intestinal cells [25]. The penetration of this layer by lipid is dependent on the incorporation of the lipid into bile salt-containing mixed micelles. Triacylglycerols and olestra are not incorporated into these micelles, presumably because of their molecular size. It therefore is very likely that the unstirred water layer will prevent an unhydrolyzed lipid from enhancing the secretion of a lipophile from enterocytes. The presence of 15 g of unhydrolyzed lipid in the intestine in the form of emulsified droplets of radius of 1 micrometer would have a surface area of 50 square meters. Although this area is large enough to interact with a part of the approximately 300 square meters of intestinal absorptive area, it should be noted that these droplets contain surfaces of bile salts and polar lipids. The presence of the unstirred water layer presumably will prevent efficient contact with the enterocyte membranes.

Finally, the effect of nonabsorbable dietary lipid on the absorption of OCs from the diet is consistent with a partitioning of OC into that lipid both in the stomach and in the intestine. OCs that enter the intestine in bile or by direct secretion from the enterocyte presumably enter the same pool of micellar and emulsified OCs as those from the diet. Nonabsorbable lipid affects both dietary and enterohepatic OCs in a similar manner in the milieu of the small intestinal lumen. 

There is, however, support for the “dialysis” mechanism for nonabsorbable lipid in the large intestine. Rozman published results that argue strongly that hexachlorobenzene (HCB) enters the intestinal tract primarily in the large intestine [14]. Rats continued to excrete HCB even when bile flow into the intestine was diverted by ligation. Monkeys with bile diversion also excreted HCB in feces. It is possible that other OCs are also excreted through secretion and desquamation into the large intestine. If this route accounts for a major part of fecal excretion, it is indeed possible that the presence of a nonabsorbable lipid in the colon may accelerate this process. The colon normally does not contain unabsorbed triacylglycerol since it is well absorbed in the small intestine. Most of the small amounts of fat that enter the colon are in the form of fatty acids and soaps after hydrolysis by pancreatic and bacterial lipases. It is possible that nonabsorbable lipid in the large intestine may facilitate the transport of OCs from intestinal cells to the lumen and thereby enhance their removal form the body in this manner. It should be noted that this mechanism does not depend on interrupting enterohepatic circulation of OCs. 

There is, however, a significant question about how unabsorbed lipid in the intestine might interact with cells in contact with the lumen of the large intestine. If the lipid is in a phase that is separate from the other components in the milieu of the large intestine, then it may indeed interact with the cells and “extract” lipophilic compounds from the membrane. If, however, the unabsorbed lipid is dispersed with the other components that comprise fecal matter, it would seem unlikely that there would be enough contact between the surface of the lipid and the cells to affect a significant extraction of cellular OCs. Nevertheless, since colonic contents do not normally contain significant amounts of lipid that might act as a solvent, the presence of unhydrolyzed fat might provide a stimulus to move lipophiles into the lumen. Moreover, as pointed out by Schlummer et al., the residence time in the large intestine is long relative to that in the small intestine, and transport from tissue to lipid-laden fecal matter may take place [26].

There is no reason to conclude that the two mechanisms of interruption of enterohepatic circulation and of enhancing secretion throughout the small and large intestine are mutually exclusive. It is possible that nonabsorbable fat acts by both mechanisms.

## 5. The Influence of Body Fat

The hypothetical calculation presented previously assumed approximately normal body weight and body fat composition. A normal body mass index (BMI) of 25 corresponds to a range of 20–25% body fat [27]. Individuals with BMI of 35 may have a body composition with 30–55% fat.

In this instance, a mass of body fat might be 40% of 70 kg, or 28,000 g. Given the same total body burden in two individuals with 14 and 28 kg of adipose tissue, the concentration in adipose in the person with twice as much fat will presumably be half of that of the leaner individual. The concentration of OCs in the blood lipids will also be correspondingly reduced. Although the total amount of blood may also be greater in the person with higher body fat, it is likely that the total amount of OC in the blood circulation will be a smaller fraction of the total body burden when the fat depot is large relative to a smaller depot. 

Support for this conclusion is seen in the study of half-lives of OCs reported by Milbrath et al. [28]. In a study summarizing reported OC half-lives, they found that the half-life of 2,3,7,8-tetrachlorodibenzo*-p-*dioxin (TCDD) increased with increasing body fat. When body fat was expressed as percent of body mass, the half-life doubled from approximately 4 to 8 years when percent body fat increased from 20 to 35%. When body fat expressed as total mass was considered, the half-life increased from 4 to 8 years as body fat increased from approximately 18 to 28 kg. Increased morbidity and decreased longevity generally observed in obese individuals may, in some cases, be related to sequelae of persistent body burdens.

Presumably the fraction of an OC stored in the body that undergoes enterohepatic circulation is a function of the fraction of the OC that is carried in the blood to the liver and enterocytes. The efficacy of an intervention that interrupts enterohepatic circulation would therefore be predicted to be related to the amount of body fat that stores the OC and limits its appearance in the blood. A high level of body fat would be expected to limit the effects of interference with enterohepatic circulation if only a small fraction of the body burden of OCs appears in the lumen of the intestine.

Vigorous exercise, sauna therapy, and supplementation with glutathione enhancers may also facilitate mobilization of toxicants from adipose storage sites [29]. Cholagogues and choleretics can also be used clinically to stimulate the secretion of toxicant-containing bile into the intestinal lumen to enhance availability for potential removal.

## 6. Weight Loss and Mobilization of Adipose Tissue

Given the indications that high BMI and body adipose depot can reduce the elimination rate of stored OCs, it is important to consider the effects of weight loss on the normal elimination rate. It is also important to consider the potential for enhancing elimination by reducing enterohepatic circulation of the OC and its lipophilic metabolites. There is much evidence that a reduction in fat stored in adipose tissue results in an increase in OC concentration in adipose tissue, and also an increase in the appearance of OC in the blood. In addition, the mobilization of OCs from adipose tissue results in their distribution into other tissues. 

We studied the effects of the interruption of enterohepatic circulation during weight loss [16]. In mice that lost weight during a regimen of caloric restriction, the concentration of hexachlorobenzene (HCB) in the brain more than tripled as adipose tissue mass decreased. In another group of calorically restricted mice that also ate olestra, the increased concentration of HCB in the brain was reduced by 50% relative to the increase in the animals that were calorically restricted without dietary olestra.

Also in that study we observed that olestra caused a dramatic increase in the fecal excretion of HCB. We later confirmed that the increase in excretion during the feeding of olestra was 25–60% greater during weight loss than during *ad lib* feeding [30]. Mutter et al. also had observed that fecal excretion of DDE in gerbils was markedly higher when olestra was fed during dietary restriction than during a period when the regimen was by dietary restriction alone [13].

Arguin et al. reported that the consumption of olestra during a 90-day weight loss regimen in humans reduced the increase in blood levels of *β*-hexachlorocyclohexane relative to that seen in during weight loss without olestra [31]. Although the relatively short duration of this trial limits conclusions about the effects of olestra, the observation is consistent with the results seen in excretion rates in mice and gerbils.

Given the continually increasing incidence of obesity seen in the United States and other developed countries, there is a high level of emphasis on the development of pharmaceuticals, dietary regimens, and surgery to reduce accumulated body fat. Diet and pharmaceuticals currently result only in modest fat reductions, but bariatric surgery has been repeatedly used to achieve large reductions in body weight and body fat. It is not clear how the rapid weight loss seen after bariatric surgery affects the distribution of OCs in patients. Some of these surgeries may result in malabsorption of OCs and interrupt enterohepatic circulation. Whether the interruption of enterohepatic circulation by other means may be of benefit in some of these cases is unknown. 

## 7. Milk

There have been numerous reports that a primary excretory route for OCs from women is in breast milk [32]. OCs are readily mobilized from adipose tissue and transported into milk lipids during lactation. Studies in rats have found similar effects. For example, a single lactation cycle depleted a dam of 98% of her body burden of 2,4,5,2′,4′,5′-hexachlorobiphenyl [33].

**Figure 2:**
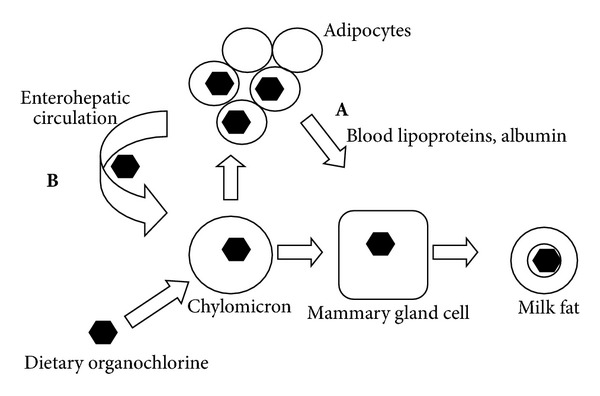
OC compounds (depicted by solid hexagons) are transported to mammary gland cells from adipose tissue carried by lipoproteins and albumin in blood (A). Chylomicrons also carry OCs from both the diet and enterohepatic circulation (B). Aim 1 addresses transport at (A).

Presumably the principal driving forces behind the appearance of OCs in milk are the concentration of the OC in adipose tissue, the mobilization of adipose tissue to provide triacylglycerol fat in the milk, and the movement of OCs with the mobilized fat to the mammary gland cells. The interruption of enterohepatic circulation of OCs would be predicted to have an effect on their appearance in milk fat primarily through a reduction in their adipose stores. A schematic view of how dietary and enterohepatic lipid and OCs can enter milk is presented in Figure 2.

It is not clear, however, if a biologically significant amount of OCs in the lumen of the intestine enters milk directly without first passing through storage in the adipocytes. There is evidence in humans that a small fraction of dietary fatty acids from the diet enters milk within a period of time that is consistent with direct entry into milk [34]. It is not known if other lipophilic nutrients follow the pattern of the fatty acids and also appear in milk soon after ingestion. If dietary OCs or OCs secreted into the intestine accompany dietary fat into milk, then it may be possible to reduce their entry into milk lipids by dietary nonabsorbable lipid or lipase inhibitor. At this time further study is needed to determine if this potential effect is clinically meaningful.

## 8. Emerging Clinical Considerations

Various medical bodies, such as the Pediatric Academic Societies, have recently concluded that “low level exposure to environmental toxicity may be impacting the functioning of the current generation [35].” With the recent emergence of abundant scientific literature correlating exposure to various toxicants with adverse clinical states and mounting awareness of the escalating chemical erosion of human health resulting from widespread bioaccumulation of chemical toxicants [36, 37], it is anticipated that intervention to diminish toxicant burdens in order to preclude and treat disease will eventually become a fundamental component of clinical medicine [38]. Numerous and varied disorders including congenital anomalies [39], neurodevelopmental conditions [40], autoimmune disorders [41], diabetes, [42], endocrine dysfunction [43], mental illness [44], cancer [45], neurodegenerative disease [46] and other assorted afflictions spanning the spectrum of medical specialties have now been directly linked, in some cases, to chemical toxicant exposures. 

While some researchers and clinicians have pursued broad-based strategies to eliminate accrued toxicants from the human body in an effort to ameliorate illness [29, 47], no single practical mechanism has yet been identified to eliminate the expansive range of persistent toxicants [38]. One of the main challenges facing researchers and clinicians, as discussed, has been the difficulty eliminating persistent lipophilic toxicants which often have prolonged half-lives because of their affinity to adipose tissue and their propensity for enterohepatic recycling. The clinical strategy of using nonabsorbable fats or other agents to impair fat absorption in conjunction with caloric restriction shows significant promise as a practical intervention to hasten the excretion of accrued lipophilic toxicants and to thus diminish the risks associated with toxicant persistence. Some concerns and novel ideas have recently emerged, however, with the clinical use of some aspects of this approach. 

The use of tetrahydrolipstatin (Orlistat)—a pancreatic lipase inhibitor which adds nonabsorbable lipid to the lumen of the intestine by inhibiting lipid absorption—has recently come under scrutiny as a result of alleged adverse effects associated with the ingestion of this drug. Although the reported incidence of serious sequelae is decidedly low in relation to the amount of product that has been used (for purposes of weight loss), recent claims about serious hepatic injury [48–51] have recently prompted the US Food and Drug Administration to issue warnings about the potential risks associated with consumption of this compound [52, 53]. This caution and the associated media attention have resulted in diminished clinical use of Orlistat for weight loss. As a result, other pancreatic lipase inhibitors are being explored [53, 54], including components of grape seed extract (GSE) [55, 56], chitosan [57], and epigallocatechin-3-gallate (EGCG) found in some teas [58, 59]. Given the limited study of these materials for detoxification purposes, however, their long-term efficacy and safety profile has yet to be determined. 

The use of olestra has been associated with gastrointestinal concerns and inhibition of fat-soluble vitamin absorption. In clinical trials, however, gastrointestinal events have been found not to differ from those experienced during consumption of foods with normal fats [60]. All olestra products are supplemented with vitamins A, D, E, and K to compensate for interference with the absorption of these nutrients. As noted below, during a one-year clinical trial testing 15 g/day of olestra in the removal of PCBs, gastrointestinal events were minimal and transient.

Acrylamide, a widespread contaminant formed in baked and fried starchy foods, is a significant component of potato chips—the primary medium currently used for therapeutic delivery of olestra. Acrylamide has evoked much attention of late with its recent classification as a “probable human carcinogen” [61]. In addition, developmental toxicity of acrylamide has been identified in human studies [62, 63], and other research suggests that exposure and potential bioaccumulation of acrylamide may also be associated with neurotoxicity [64] as well as genomic, hormonal, and testicular dysfunction in animals [65]. The use of potato chips as a delivery vehicle for olestra will not add to acrylamide ingestion when the chips are intentionally substituted for other baked and fried carbohydrate-based foods containing acrylamide. Other mediums to deliver olestra, however, may be considered in order to preclude this exposure.

There is also ongoing exploration of other lipid compounds that are not well absorbed and which might interrupt enterohepatic recycling of lipophilic compounds. Castor oil, for example, has been found to be therapeutic in some situations of toxicant overdose [66] while mineral oil has been noted to enhance fecal excretion of lipophilic DDT [67]. In review, the data suggests that clinical strategies to mobilize toxicants (such as caloric restriction) in combination with the provision of nonabsorbable lipid to the intestinal lumen may be useful in diminishing body burdens of OC compounds. Research is underway at various centers to explore safe and practical strategies that might have a clinical role in facilitating human elimination of lipophilic and other toxicants.

## 9. Studies in Progress

A one-year study of the effect of olestra on the body burden of PCBs in a cohort with elevated PCB levels has been completed. Blood levels of PCBs were measured during the trial, and the data are being evaluated. The trial began with fourteen participants in the control group receiving potato crisps made with vegetable oil, and fourteen in the olestra group receiving potato crisps made with olestra. The olestra dose was 15 g/day. Twelve participants in the control group completed the trial, and eleven, in the olestra group. Adverse events were modest and transient, with only one dropout reporting gastrointestinal problems, and that person received the vegetable oil crisps. This trial is registered in http://ClinicalTrials.gov/, with registration number NCT01261338.

Another study has been designed to determine if castor oil along with lipase inhibitors GSE and EGCG, in combination with other binders including pectin [68], clay [69], and activated charcoal [70] might be of clinical use. Taken after tissue toxicant mobilization and use of modalities to enhance biliary secretion into the small intestine, it is to be determined if this protocol used intermittently might facilitate the elimination of a broad range of adverse compounds including OCs. Chronically ill individuals with varying types of toxicant burdens will use this protocol, with pre- and posttreatment stool collections to be evaluated for comparative levels of a multiplicity of recognized toxicants. 

## 10. Conclusion

The addition of nonabsorbable lipid to the intestinal lumen interferes with the absorption of OCs from the diet by sequestering the OC in the lipid phase and transporting it to the colon and feces. This nonabsorbable lipid phase also interferes with the reabsorption of OCs that enter the intestinal lumen in bile or through enterocyte secretion or sloughing. There is a possibility that the lipid phase enhances this transport from the enterocyte, but more study is needed to confirm this possibility. Weight loss mobilizes OCs from adipose tissue, and enhances their appearance in blood and intestine, and thereby increases the possibility for interaction with nonabsorbable lipid in the intestine. Nonabsorbable lipid might affect the appearance of OCs in milk, but there currently are no data to support or refute this possibility. 

It is possible to add nonabsorbable lipid to the lumen of the intestine, either as a dietary additive such as olestra or through the inhibition of lipase by a lipase inhibitor such as tetrahydrolipstatin. Established risks associated with either regimen are thus far limited to reductions in absorption of fat-soluble vitamins and loose stools which can often be moderated by the adjustment of dose. As of this time, our understanding is that the risk associated with olestra is small-based, in part, on outcomes of a one-year trial in subjects exposed to PCBs. The clinical use of nonabsorbable lipid on a long-term basis should, however, be dictated by the consideration of risk and benefit. Clinical strategies to mobilize toxicants (such as caloric restriction) in combination with the provision of nonabsorbable lipid to the intestinal lumen may be useful in diminishing body burdens of OC and other lipophilic compounds.
